# Overlap Chronic Placental Inflammation Is Associated with a Unique Gene Expression Pattern

**DOI:** 10.1371/journal.pone.0133738

**Published:** 2015-07-24

**Authors:** Kripa Raman, Huaqing Wang, Michael J. Troncone, Waliul I. Khan, Guillaume Pare, Jefferson Terry

**Affiliations:** 1 Population Health Research Institute, Hamilton Health Sciences and McMaster University, Hamilton, Ontario, Canada; 2 Thrombosis and Atherosclerosis Research Institute, Hamilton Health Sciences and McMaster University, Hamilton, Ontario, Canada; 3 Farncombe Family Digestive Health Research Institute, Hamilton Health Sciences and McMaster University, Hamilton, Ontario, Canada; 4 Department of Pathology and Molecular Medicine, Hamilton Health Sciences and McMaster University, Hamilton, Ontario, Canada; Xavier Bichat Medical School, INSERM-CNRS - Université Paris Diderot, FRANCE

## Abstract

Breakdown of the balance between maternal pro- and anti-inflammatory pathways is thought to allow an anti-fetal maternal immune response that underlies development of chronic placental inflammation. Chronic placental inflammation is manifested by the influx of maternal inflammatory cells, including lymphocytes, histiocytes, and plasma cells, into the placental membranes, villi, and decidua. These infiltrates are recognized pathologically as chronic chorioamnionitis, chronic villitis of unknown etiology, and chronic deciduitis. Each of these histological entities is associated with adverse fetal outcomes including intrauterine growth restriction and preterm birth. Studying the gene expression patterns in chronically inflamed placenta, particularly when overlapping histologies are present, may lead to a better understanding of the underlying mechanism(s). Therefore, this study compared tissue with and without chronic placental inflammation, manifested as overlapping chronic chorioamnionitis, chronic villitis of unknown etiology, and chronic deciduitis. RNA expression profiling was conducted on formalin fixed, paraffin embedded placental tissue using Illumina microarrays. *IGJ* was the most significant differentially expressed gene identified and had increased expression in the inflamed tissue. In addition, *IGLL1*, *CXCL13*, *CD27*, *CXCL9*, *ICOS*, and *KLRC1* had increased expression in the inflamed placental samples. These differentially expressed genes are associated with T follicular helper cells, natural killer cells, and B cells. Furthermore, these genes differ from those typically associated with the individual components of chronic placental inflammation, such as chronic villitis, suggesting that the inflammatory infiltrate associated with overlapping chronic chorioamnionitis, chronic villitis of unknown etiology, and chronic deciduitis differs is unique. To further explore and validate gene expression findings, we conducted immunohistochemical assessment of protein level expression and demonstrate that IgJ expression was largely attributable to the presence of plasma cells as part of chronic deciduitis and that IgA positive plasma cells are associated with chronic deciduitis occurring in combination with chronic chorioamnionitis and chronic villitis of unknown etiology but not with isolated chronic deciduitis.

## Introduction

During pregnancy the maternal immune system recognizes paternal alloantigens expressed by the fetus but typically does not generate a significant anti-fetal inflammatory response. Breakdown of the balance between pro- and anti-inflammatory pathways involved in maternal tolerance is thought to permit an anti-fetal maternal immune response that has been likened to allograft rejection [1, 2]. Mechanisms that protect the fetus from an aberrant maternal immune response are currently being defined but the exact nature of this process, and why it occasionally fails, remains unclear [22]. What is becoming apparent is that interaction between the placenta, decidua, and immune effectors at the fetomaternal interface are involved in the maintenance of tolerance. Furthermore, appropriate regulation of T cell function is an important factor [3, 4].

Loss of maternal tolerance to fetal tissue engenders an inflammatory response comprised primarily of T cells, histiocytes, and plasma cells. This cellular response is evident upon histopathological examination of affected placental and decidual tissue as chronic chorioamnionitis (CC), chronic villitis of unknown etiology (VUE), and chronic deciduitis (CD) [4–6]. Cumulatively these histological entities are forms of chronic placental inflammation (CPI) and can be associated with adverse fetal outcomes such as stillbirth, intrauterine growth restriction, preterm labor, spontaneous abortion, and neurological impairment [5–7]. Investigations into the pathogenesis of CPI typically focus on a single histological entity, however these studies may not be representative of the subset of cases where an overlap of more than one histological pattern of chronic inflammation is present. Assessment of tissue with CC, VUE, and CD together may reveal unique inflammatory features and provide additional clues to the mechanism(s) underlying the development of overlap CPI (oCPI).

One method to explore the differences between oCPI and non-inflamed control tissue is through gene expression analysis. Selection of fresh oCPI tissue for gene expression analysis is problematic as chronic inflammation is spatially and temporally variable and typically is not evident during standard gross placental examination. The use of formalin fixed, paraffin embedded (FFPE) tissue for gene expression study of oCPI is preferable as it allows positive selection of chronically inflamed tissue and incorporation of tissue with similar spatial and temporal distributions of inflammation. The use of FFPE tissue also facilitates correlation between histopathological, immunophenotypic, and gene expression data. Despite the many potential benefits of FFPE tissue, the increased degradation of mRNA in archival tissue has historically restricted its use in gene expression studies. However, the complementary DNA-mediated Annealing, extension, Selection and Ligation (DASL) assay (Illumina), is an expression profiling method suitable for use with degraded RNA. In the DASL assay, cDNA synthesis is conducted using both oligo(dT) and random primers, therefore facilitating amplification of partially degraded RNA species. Gene probes for the DASL assay span ~50 bases, also enabling identification of partially degraded transcripts. Furthermore, studies have shown that expression profiles of FFPE tissue are comparable to expression profiles of fresh frozen tissue when using the DASL assay [8]. As such, in this work we assess gene expression patterns of archival chronically inflamed placental tissue selected specifically to include CC, VUE and CD. Using the DASL assay we identify genes differentially expressed between placentae samples with and without chronic inflammation. We also explore the protein-level implication of the most significant gene using immunohistochemical studies.

## Materials and Methods

### Tissue Selection

Ethics approval was provided and the need for consent waived by the Human Tissue Subcommittee of the Hamilton Integrated Research Ethics Board for an anonymized human tissue study in accordance with the Canadian Tri-Council policy statement on ethical conduct for research involving humans prior to tissue retrieval (HIREB project number 13-071-T). The criteria for placental pathology are defined as previously described [5, 9]. For the purposes of this study, oCPI is defined as the presence of CC, VUE, and CD. The oCPI placental blocks were selected to include CC, VUE, and CD with an approximately similar proportion of chronically inflamed villi and adherent decidua between cases. The oCPI cases were not selected or stratified based on grade of VUE or chronic chorioamnionitis. However, the grade of VUE corresponded to low grade, multifocal in 4 cases and high grade, patchy in 2 cases as assessed according the scheme proposed by Redline [10]. All oCPI cases were stage 1, grade 1 according to the CC grading scheme proposed by Kim et al. [5]. OCPI cases were excluded if the following were present: acute placental inflammation, intervillus histiocytosis, an identified or suggested infectious cause in the mother or fetus, or other histopathological placental abnormality. Gestational age for the oCPI cases ranged from 33 to 38 weeks and all placental weights were below the 10th percentile for gestational age [11]. Control tissue was collected from cases of uteroplacental underperfusion (UPU) without additional diagnostic placental abnormalities. The selection of gestational age matched, third trimester, non-inflamed UPU placenta as control tissue was sought to minimize bias due to gestational-age related changes in placental and decidual tissues [2, 12]. UPU controls were matched to the oCPI cases for trimmed placental disc weight within 10% and gestational age at delivery within 7 days. Control UPU blocks lacked areas of infarction, maternal decidual vasculopathy, and inflammatory infiltrates aside from decidual lymphocytes. All samples were selected from placentas submitted within the previous 3 years (2011–2013) to minimize the potential negative effect of specimen storage time [13]. Using these selection criteria 6 cases of oCPI and 5 control cases are obtained from the archives of McMaster Children’s Hospital for RNA extraction. Six isolated CD cases for immunostaining were derived from placenta with CD but no histological evidence of VUE or CC.

### RNA Isolation

Total RNA was isolated from five 10-micron tissue sections from 2 separate areas of placental disc (100 microns total placental tissue). Extraneous paraffin was manually removed and the isolated tissue was combined. Total RNA was isolated using the High Pure RNA Paraffin Kit (Roche, Mississauga, ON). Deparaffinization, purification, DNase incubation, Proteinase K treatment, and other steps were performed according to the manufacturer’s protocol. RNA quantity was determined using RiboGreen (Life Technologies, Burlington, ON), while quality was verified primarily using Nanodrop spectroscopy (Thermo Fisher Scientific, Wilmington, DE). In a subset of samples (3 total) quality was also assessed using the Agilent 2100 Bioanalyzer. Total RNA samples were stored at -80°C until further processing.

### Microarray Hybridization

For the microarray analysis, 200 ng of total RNA was converted to cDNA and biotin labeled using the whole genome cDNA mediated annealing, selection, extension, and ligation assay (WG-DASL) assay according to the manufacturer’s protocol (Illumina, San Diego, CA). Samples were then hybridized to the Illumina HumanRef-8 v4 BeadChip (Illumina) which interrogates 29 285 RNA transcripts. The chips were washed, dried, and imaged on the iScan System (Illumina).

### Data Pre-Processing

The sample probe profile and control probe profile were output from GenomeStudio (Illumina) without background correction [14]. The original data files have been deposited in Gene Expression Omnibus (GEO, http://www.ncbi.nlm.nih.gov/geo) and are accessible through GEO Series accession number GSE68474. All quality control and data preprocessing was performed in R, a statistical analysis program, and using microarray-specific packages available through Bioconductor, lumi and LIMMA [15, 16]. Quality control involved assessment of raw expression boxplots, interquartile range, Pearson correlation between samples and density plots; outlier samples were not identified. Data pre-processing consisted of background correction using the non-genomic control probes on each chip followed by quantile normalization and log2 transformation [17]. Probe filtering was conducted using detection P-value, the probability of a false positive signal calculated based on the background signal from the non-genomic negative control probes. Probes were considered expressed if they had a detection P-value <0.01 in at least half of the samples. The resulting pre-processed expression matrix consisted of 21 172 probes.

### Statistical Analysis

Unsupervised hierarchical clustering and heat maps were generated using R and Bioconductor packages. Bayesian moderated t-tests were used to identify differentially expressed probes between inflamed placentae and controls. To minimize false positives and correct for multiple hypothesis testing, an adjusted P-value was calculated using the Benjamini-Hochberg false discovery rate (FDR) correction. An FDR P-value < 0.05 was considered significant. Unpaired double-tailed Student’s t-test was used to compare the relative abundance of IgA, IgM, and IgG positive plasma cells.

### Immunohistochemistry

Immunohistochemical studies were performed on 4 micron tissue sections. IgJ immunostaining was performed manually with sodium citrate buffer heat-induced epitope retrieval, a 1/100 dilution of rabbit anti-human IgJ monoclonal antibody (clone SP105, Abcam, Toronto, ON; Antibody registry ID AB_10902174), and Envision secondary antibody (Dako, Burlington, ON). Immunohistochemistry for IgA, IgG, IgM, CD10, CD34, TdT, and PAX5 was performed using standard clinical antibodies and automated protocols on a Ventana Benchmark XT automated immunostainer (Tucson, AZ, USA). Normal tonsil and bone marrow was used as positive controls to demonstrate appropriate immunostaining patterns for all antibodies. Negative controls involved omission of primary antibody and showed negligible background staining.

## Results

### Quality Control Data

**Fig 1:**
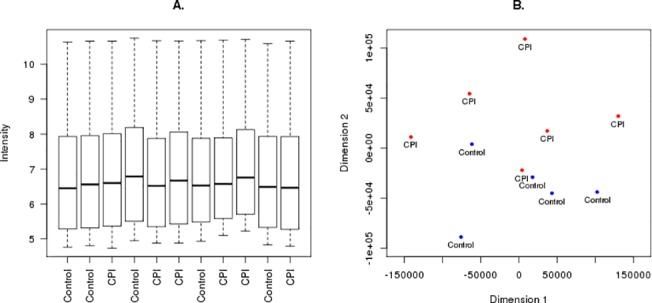
(A) Boxplots of raw microarray intensity values demonstrate little variation between samples. (B) Multidimensional scaling (MDS) plot of raw intensity values, which visualizes the similarity between samples and demonstrates separation between the oCPI and control samples.

We extracted total RNA from 11 FFPE tissue blocks. The total RNA yields from FFPE were variable, ranging from 4 to 20 ng/uL, and did not correlate with the amount or age of samples. Nanodrop spectroscopy readings indicated that the RNA was of good quality, RNA 260/280 > 1.8. Bioanalyzer tracings demonstrated that the RNA was degraded as expected with the majority of RNA transcripts ~200bp in length and RNA integrity number (RIN) values below 2. It is important to recognize that the RIN is a semiquantitative predictor of interexperimental variability for a particular methodology but is not a robust predictor of successful experimental outcomes as this is dependent on the methodology employed [18]. In this respect, the DASL method is specifically designed to amplify short (50 bp) RNA fragments and as such a RIN value < 2 with average RNA fragment lengths of 200 bp suggests suitable RNA integrity for this method [19]. The RNA isolated from all archival samples in this study is of adequate purity and average fragment length to proceed with WG-DASL gene expression analysis. Visual inspection of the microarray chips did not suggest spatial artifacts or abnormalities (data not shown). Boxplots of the log2 transformed intensity values showed that the quality of the data did not vary between samples (Fig 1A). Furthermore multidimensional scaling (MDS) plots, an unbiased method to visually identify similarities between samples, suggested biologically relevant separation between oCPI samples and controls (Fig 1B).

### Differential Expression Analysis

**Fig 2:**
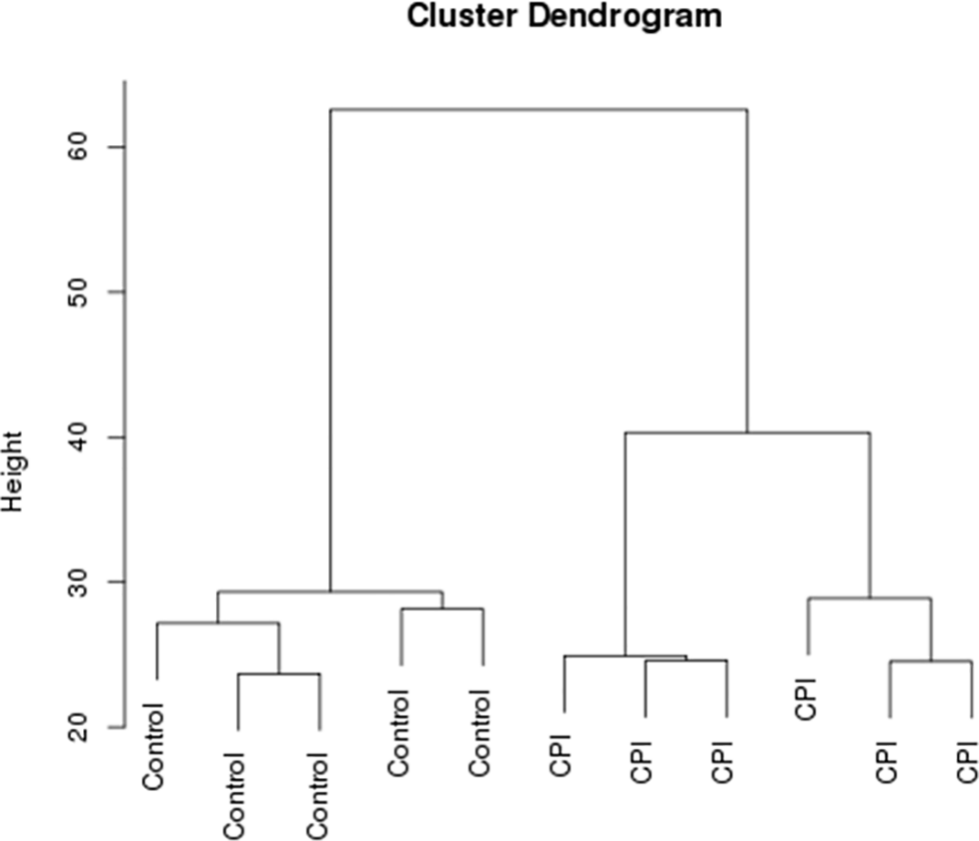
Unsupervised hierarchical clustering, using the 1446 probes with suggestive differential expression (P-value < 0.05) between oCPI samples and controls, demonstrates complete separation of the two groups.

**Fig 3:**
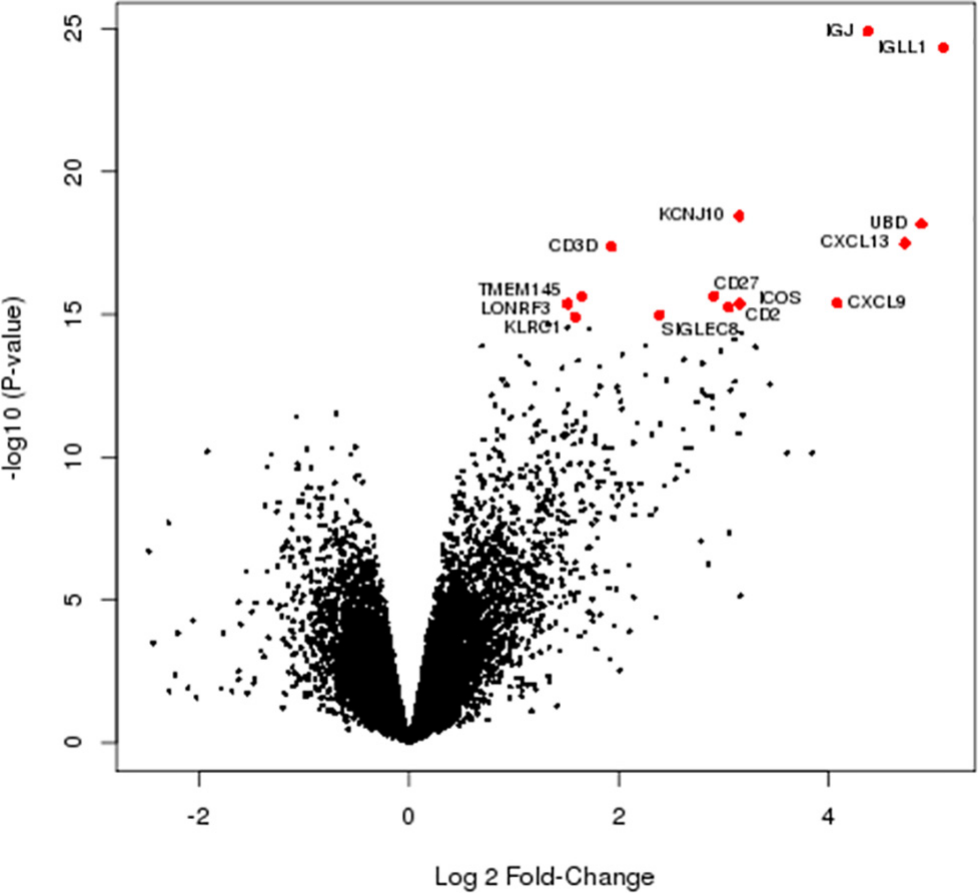
Volcano plot of expression association with oCPI. Each dot represents one of the 21 172 probes. The x-axis represents the effect of each gene, reported as log2 fold change, while the y-axis represents the –log10 (P-value). A positive log2 fold change is indicative of increased expression in oCPI samples as compared to controls. The red dots highlight the 14 genes with significant differential expression (FDR P-value < 0.05).

Using the Bayesian-moderated t-test we identified 1446 RNA transcripts with suggestive differential expression, corresponding to 1293 unique genes, that were differentially expressed between oCPI and control samples (P-value <0.05). Unsupervised clustering using only these transcripts demonstrates complete separation of the oCPI samples from the non-inflamed control samples (Fig 2). 64% of the probes (N = 928) showed increased expression in the oCPI samples, which is visualized in a volcano plot (Fig 3). After FDR correction for multiple hypotheses testing only 14 unique genes remained significantly associated with oCPI (Table 1). Each of these genes displayed increased expression in oCPI samples as compared to controls (Fig 3). Biologically these genes have been associated with development of immune tolerance and memory (*ICOS* [20], *CD27* [21]), T cell activation (*CXCL13*, *CXCL9* [22]), NK cell activation (*KLRC1* [23]), B cell homing (*CXCL13* [24]), immunoglobulin production (*IGJ* [25]), B cell development (*IGJ* [26], *IGLL1* [27]), decidual regulation of inflammation (*ICOS* [28], *CXCL13* [29]), and suppression of eosinophilic inflammation (*SIGLEC8* [30]). Although the 14 oCPI-associated genes are not specifically indicative of a molecular process or abnormality, together they are reminiscent of genes involved in germinal center function with a focus on follicular helper T cells (T_FH_) cells [31].

**Table 1:** RNA transcripts significantly associated with oCPI. 14 differentially expressed transcripts were identified using linear models (FDR P-value<0.05). A positive fold change indicates increased expression in oCPI samples as compared to controls.

Gene	Fold change	P-value	FDR P-value	Description
**IGJ**	20.8	3.17 x 10^−8^	5.01 x 10^−4^	Immunoglobulin joining chain
**IGLL1**	34.2	4.73 x 10^−8^	5.01 x 10^−4^	Immunoglobulin lambda-like polypeptide 1
**KCNJ10**	8.9	2.80 x 10^−6^	0.018	Potassium inwardly-rectifying channel, subfamily J, member 10
**UBD**	29.6	3.41 x 10^−6^	0.018	Ubiquitin D
**CXCL13**	26.5	5.44 x 10^−6^	0.021	Chemokine
**CD3D**	3.8	5.89 x 10^−6^	0.021	Delta component of T-cell receptor
**CD27**	7.5	1.96 x 10^−5^	0.046	TNF receptor
**TMEM145**	3.1	1.98 x 10^−5^	0.046	Transmembrane protein
**CXCL9**	16.9	2.31 x 10^−5^	0.046	Chemokine
**ICOS**	8.9	2.35 x 10^−5^	0.046	Inducible T-cell co-stimulator
**LONRF3**	2.9	2.37 x 10^−5^	0.046	LON peptidase N-terminal domain and ring finger 3
**CD2**	8.3	2.59 x 10^−5^	0.046	CD2 molecule
**SIGLEC8**	5.2	3.12 x 10^−5^	0.049	Sialic acid binding Ig-like lectin 8
**KLRC1**	3.0	3.26 x 10^−5^	0.049	Killer cell lectin-like receptor subfamily C, member 1

### Localization of IgJ Expression in oCPI Tissue

*IGJ* was the most significant gene identified with increased expression in oCPI samples as compared to controls and had the most significant detection p-value in all samples (mean 0.01223 vs. 0.04639, IGJ and IGLL1 respectively). As such IGJ localization was further explored using immunohistochemistry. The most reasonable explanation for the observed increase in *IGJ* is IgM and/or IgA producing plasma cells in oCPI cases with CD. However, associated overexpression of immunoglobulin heavy chain was not observed, thus suggesting that *IGJ* overexpression may be occurring in isolation. Isolated increased expression of *IGJ* occurs early in B cell development [26], and the presence of significantly increased expression of *IGLL1* suggests that *IGJ* overexpression in the CPI group may be related to the presence of immature B cells. In addition, immunohistochemical detection of IgJ expression has been reported in Hofbauer cells in second trimester placenta [32]. To further unravel the significance of IgJ we conducted immunostaining on the oCPI and control placental disc samples initially used for gene expression analysis.

**Fig 4:**
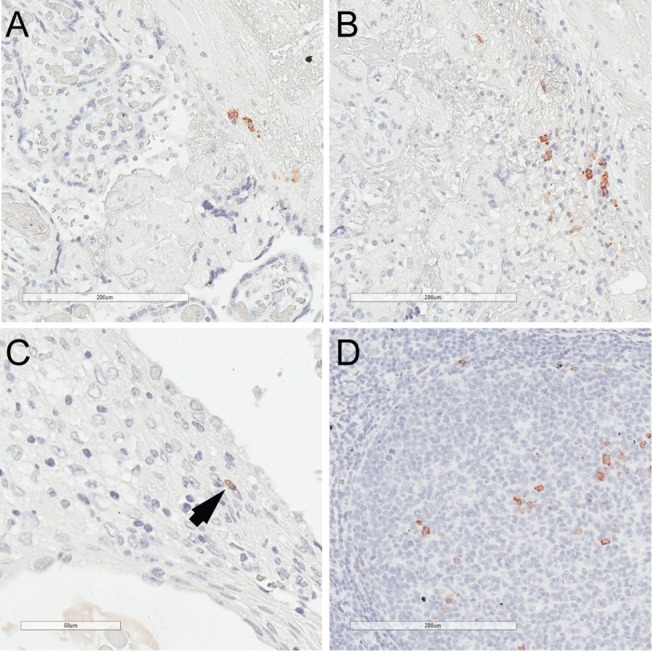
(A) Immunostaining for IgJ demonstrates reactivity in plasma cells in areas of chronic villitis and (B) chronic deciduitis (original magnifications 200x). (C) Very rare lymphocytic-appearing cells expressing IgJ were also seen (original magnification 400x). (D) Positive control tissue exhibited an appropriate IgJ staining pattern (original magnification 200x).

**Fig 5:**
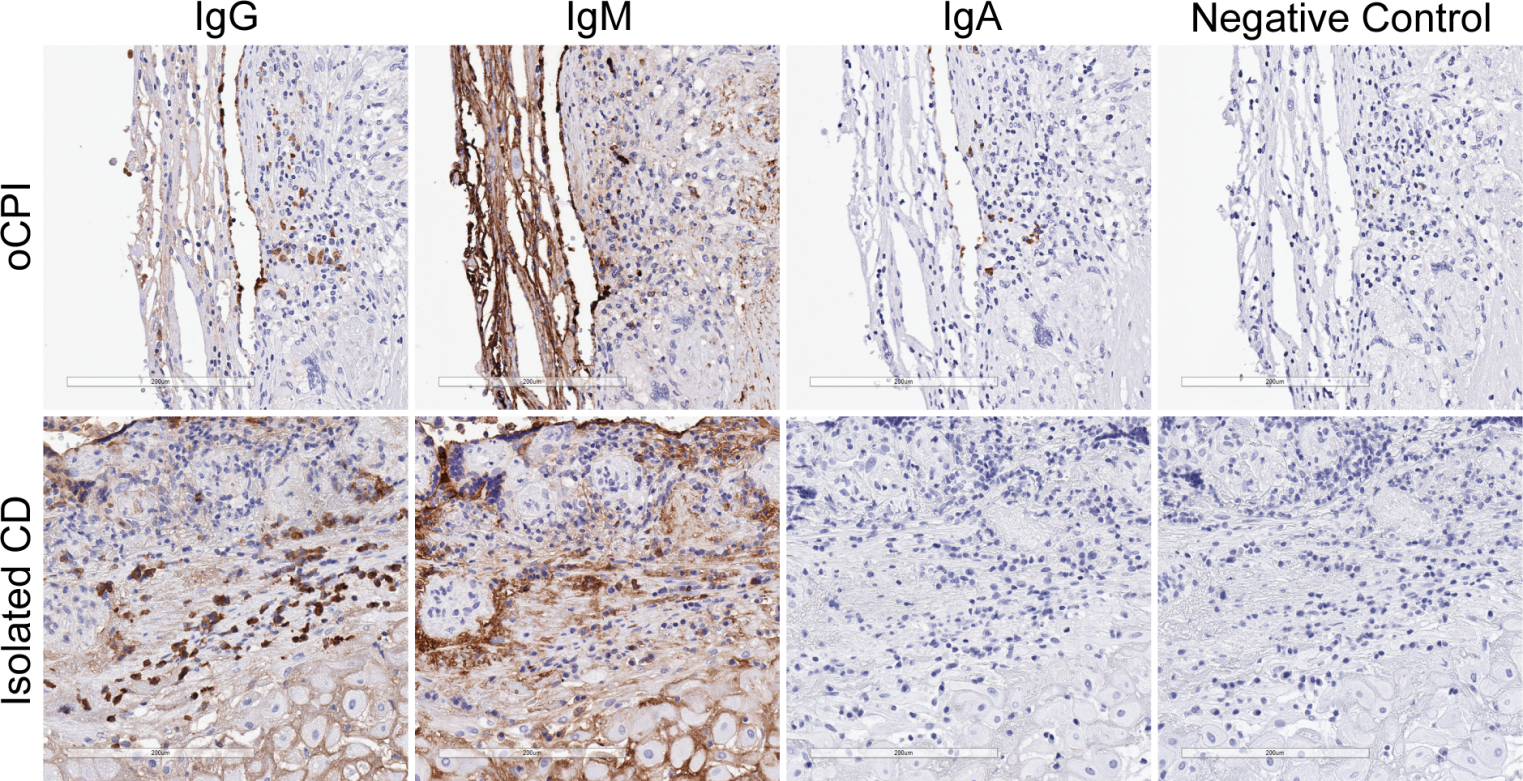
Immunohistochemical subtyping of oCPI placenta demonstrates an IgG, IgA, and IgM expressing plasma cells (top row, original magnification 200x). In cases of isolated chronic deciduitis (chronic deciduitis without histological evidence of VUE and chronic chorioamnionitis), IgG-positive plasma cells predominate, IgM-positive plasma cells are rare, and IgA-positive plasma cells are not seen (bottom row, original magnification 200x).

**Fig 6:**
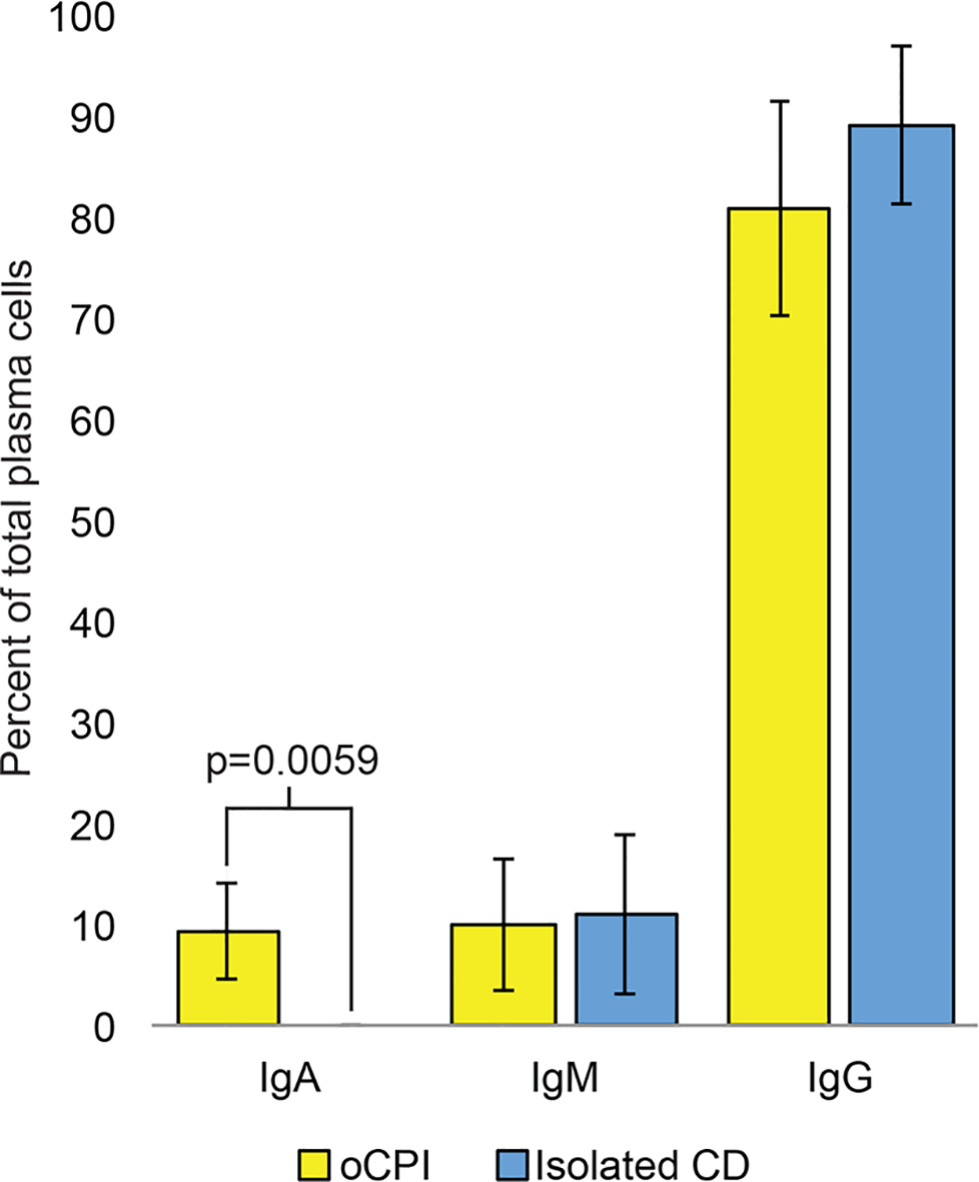
Proportions of IgA, IgM, and IgG expressing plasma cells in oCPI-related CD compared to isolated CD. Error bars represent the 95% confidence interval of the mean.

We identified IgJ protein expression by immunohistochemistry in plasma cells in areas of oCPI related VUE (Fig 4A) and CD (Fig 4B). Since IgJ comprises part of the secretory immunoglobulin IgA and the expression profile associated with oCPI includes genes related to hollow viscus immunity, we surmised a potential connection between oCPI and IgA. To investigate this further, we subclassified CD-associated plasma cells by immunohistochemistry with respect to IgA, IgM, and IgG expression. IgA positive plasma cells were present in oCPI-related CD whereas they appeared to be absent in isolated CD (i.e. without histological evidence of VUE and/or chronic chorioamnionitis) (Fig 5). This difference was confirmed by quantification of IgA, IgM, and IgG expressing plasma cells which revealed that approximately 10% of plasma cells in oCPI-related CD express IgA compared to an absence of IgA-positive plasma cells in isolated CD (Fig 6). Levels of IgG and IgM-positive plasma cells were similar in both oCPI and isolated CD.

**Fig 7:**
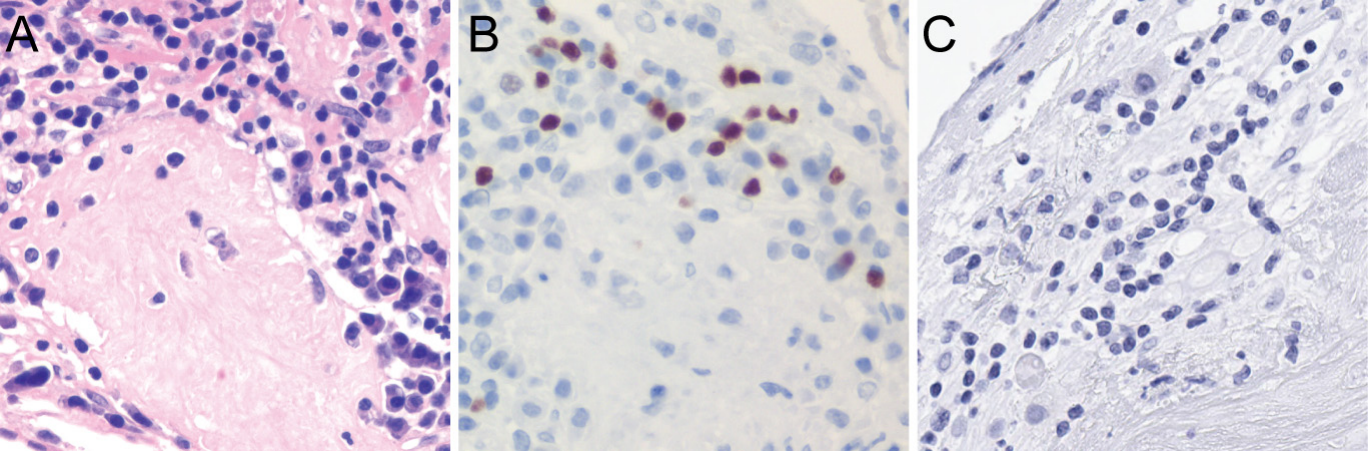
(A) Lymphocytes present as part of oCPI-related CD. (B) These lymphocytes express PAX5 consistent with B cells. (C) Immunostaining for markers of immature B cells, including TdT as shown here, are negative. All original magnifications 400x.

Very rare IgJ positive lymphocytic cells were also observed in in the decidua of oCPI placenta, suggesting the possibility of immature *IGJ* expressing lymphocytes (Fig 4C). PAX5 positive lymphocytes were focally abundant in areas of CD (Fig 7A and 7B) but correlative immunostaining of these IgJ expressing lymphocytic cells for markers of immature B-cells, including CD10, CD34, and TdT were negative (Fig 7C). Co-expression of IgJ in these B cells could not be definitively confirmed due to their relative rarity. Weak expression of IgM was noted in smaller lymphocytic cells, which may explain the associated with IgJ expression. Finally, IgJ expression was not seen in Hofbauer cells as previously reported. These findings indicate that the majority of IgJ expression in oCPI placenta is related to the presence of IgM and IgA expressing plasma cells and IgA positive plasma cells are associated with oCPI compared to isolated CD. IgJ expression does not appear to be related to the presence of immature B cells in oCPI.

## Discussion

The pathogenetic mechanism(s) underlying development of chronic placental inflammation, particularly when CC, VUE, and CD are all present, is not well characterized. This study was designed primarily to assess the gene expression pattern in tissue with oCPI to better define the nature of the associated inflammatory infiltrate and improve understanding of this process. We identified 14 genes capable of distinctly differentiating between oCPI and control samples. Each of these genes are involved in inflammation and showed increased expression in oCPI samples. Due to the variability and limited quantity of RNA extracted from FFPE placentae samples, microarray validation with RT-PCR was not possible. As such, immunohistochemistry was employed to validate some of the gene expression findings. In addition, some of the genes with increased expression in oCPI samples, such as *ICOS* and *CD27*, have been previously implicated in regulation of immune tolerance at the fetomaternal interface [22]. The concordance between our study and previous works adds further biological validity to our findings.

It is becoming increasingly evident that T-cell signaling and regulatory pathways involved in normal pregnancy and chronic placental inflammation are complex and may not follow paradigms based on findings in lymphoid tissue elsewhere [33]. Studies of the components of oCPI, such as chronic villitis, have reported an imbalance towards cellular immunity implying this as the underlying mechanism of chronic placental inflammation [22]; however, the constellation of overexpressed gene related to oCPI identified in the present study are more in keeping with a humoral-type response. These apparently contradictory findings seem to support our hypothesis that the underlying pathogenetic mechanism of oCPI differs from that of its isolated component parts; however, the possibility that these differences are methodological needs to be considered.

The observation that oCPI-related CD includes IgA positive plasma cells when compared to isolated CD provides further support for the hypothesis that oCPI is more than the sum of its histological components. The pathogenetic significance of this observation is unclear, but the role of IgA in body cavity immunity is well established and raises the possibility that oCPI represents a similar type of immunological reaction. Interestingly *CXCL13*, which is highly expressed in oCPI placenta, has also been implicated in the development of body cavity immunity [24]. This remains as a promising avenue for further investigation.

Direct comparison of our results to previous findings is hindered by the absence of studies using archival tissue specifically including CC, VUE, and CD. Our study does not stratify samples based on grades of VUE and chronic chorioamnionitis as has been done in other studies focused on these entities. This was, in part, due to the heterogeneity and subjectivity of proposed VUE grading schemes [7] and the consistent grade of chronic chorioamnionitis. Undetectable transcripts related to use of archival tissue in our study and sampling of non-inflamed or unrelated lesional tissue in fresh frozen tissue-based studies are other potential methodological explanations. Regardless, the possibility remains that the gene expression pattern demonstrated here represents a unique pathogenetic process that is correlated with the coordinated histological appearance of CC, VUE, and CD. Ultimately direct comparative studies between oCPI and isolated VUE, isolated CC, and isolated CD using the same experimental methodology is required to address these possibilities.

The involvement of T_FH_ cells in chronic placental inflammation is an intriguing possibility. *ICOS* is involved in the development of T_FH_ cells and the ICOS-B7h signaling pathway appears to play an important role in fetal-maternal tolerance by suppressing a T_h1_ type response, although the exact mechanism(s) involved remains unclear [20, 28, 33]. T_FH_ cells are vital in the development of a robust B-cell response to T-cell dependent antigens, therefore a role for T_FH_ cell dysregulation in the development of an antibody-mediated response to fetal alloantigens can be easily imagined. T_FH_ cell development is dependent on a balance of positive and negative regulatory pathways and imbalances in these signals may underlie the development of abnormal immune response-related disease [31]. This in turn suggests a potential role for genetic background influencing T_FH_ cell dysregulation in oCPI. What is harder to encompass is the localization of T_FH_ cells in oCPI since germinal center formation is not present in CPI and was not identified in any of the placental tissue in this study. This incongruity suggests that the inflammatory infiltrate associated with oCPI may not contain T_FH_ cells but other T cell subsets expressing these markers [31]; although unlikely, migrating T_FH_ cells is another possibility. A detailed assessment of the subsets of T cells present in oCPI, perhaps by flow cytometric and immunohistochemical studies, is required to better characterize the potential role of T_FH_ cells in this process.
